# A simple and rapid chromatographic method to determine unauthorized basic colorants (rhodamine B, auramine O, and pararosaniline) in processed foods

**DOI:** 10.1002/fsn3.127

**Published:** 2014-06-02

**Authors:** Chiye Tatebe, Xining Zhong, Takashi Ohtsuki, Hiroki Kubota, Kyoko Sato, Hiroshi Akiyama

**Affiliations:** National Institute of Health Sciences1-18-1, Kamiyoga, Tokyo, Setagaya-ku, 158-8501, Japan

**Keywords:** Auramine O, HPLC, pararosaniline, rhodamine B

## Abstract

A simple and rapid high-performance liquid chromatography (HPLC) method to determine basic colorants such as pararosaniline (PA), auramine O (AO), and rhodamine B (RB) in various processed foods was developed. Linearity of the calibration curves ranged from 0.05 to 50 *μ*g/mL for PA and 0.05–100 *μ*g/mL for AO and RB. The detection and quantification limits (LOD and LOQ) of the basic colorants, which were evaluated as signal-to-noise ratios of 3 for LOD and 10 for LOQ, ranged from 0.0125 to 0.05 and 0.025 to 0.125 *μ*g/g, respectively. The recoveries and relative standard deviations of three basic colorants in six processed foods, namely, chili sauce, curry paste, gochujang (hot pepper paste), tandoori chicken (roasted chicken prepared with yogurt and spices), powder soup, and shrimp powder ranged from 70.2% to 102.8% and 0.8% to 8.0%, respectively. The intraday precision of the recovery test ranged from 1.7% to 4.5%, whereas the interday precision ranged from 3.7% to 7.7%. The reported method has been successfully applied to basic colorant determination in various processed foods such as fat-based food matrices (curry paste and tandoori chicken), chili products (gochujang and chili sauce), and protein-based products (shrimp powder and powder soup). Thin layer chromatography and liquid chromatography/mass spectrometry methods for the determination of basic colorants in processed foods were also developed for rapid analysis and identification, respectively. These methods are very useful for monitoring unauthorized basic colorants in inspection centers or quarantine laboratories in many countries.

## Introduction

Synthetic food colors are used worldwide to avoid the loss of original color in processed foods, as well as to make the products more attractive to consumers. Synthetic food colors are considered superior to natural food colors in terms of their color value, uniformity, and applicability in various processed foods. Synthetic food colors have been authorized and regulated for use in food additives in many countries (Ministry of Health, Labour and Welfare 1947; European Council 1994; U.S. Food and Drug Administration 2004).

**Figure 1:**
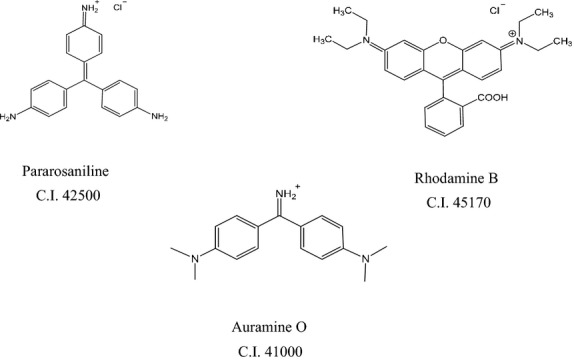
Chemical structures of PA, AO, and RB.

While the use of water-soluble synthetic acid colors such as erythrosine, amaranth, or acid red is generally allowed worldwide, some basic colorants such as pararosaniline (PA), auramine O (AO), and rhodamine B (RB) are unauthorized food additives in Japan, the EU, and the United States because of their toxicity. PA and AO are possibly carcinogenic to humans and classified into Group 2B by the International Agency of Research on Cancer (2010). Rhodamine B is also proved to be carcinogenic and toxic to humans and animals (International Agency for Research on Cancer 1978) (Fig. 1).

However, these basic colorants have been detected in various processed foods. The use of RB and AO has been reported in several developing countries such as Malaysia (Food Safety Net 2010), the Philippines (Republic of the Philippines, Food and Drug Administration 2013), India (Dixit et al. 2011; Gresshma and Reject Paul 2012), Vietnam (Sai Gon Giai Phong 2012), Argentina (Alesso et al. 2012), and China (The Government of the Hong Kong Special Administrative Region, Centre for Food Safety 2012; SGS Hong Kong Limited 2011). In Japan, the use of RB and PA in imported processed foods has also been reported (Suzuki et al. 2007; Ministry of Health, Labour and Welfare 2005). Thus, effective monitoring of basic color contaminants in processed foods is necessary to ensure food safety.

Although various analytical methods have been developed for the detection and determination of basic colorants in processed foods (Botek et al. 2007; Dixit et al. 2011; Alesso et al. 2012; Gresshma and Reject Paul 2012), such methods have several drawbacks, including time-consuming steps, a lack of application data to various processed foods, or unavailable data for the determination of low levels of basic colors. Many of them were determination methods of only RB (Alesso et al. 2012; Gresshma and Reject Paul 2012); nevertheless PA and AO were detected in processed foods as contaminants (Suzuki et al. 2007; Dixit et al. 2011), there is no simultaneous determination method of PA, AO, and RB. Therefore, it is necessary to develop a simultaneous determination method for PA, AO, and RB in various processed foods.

In this study, we developed a simple and rapid extraction and determination method to detect low levels of basic colorants (0.5 *μ*g/g) by high-performance liquid chromatography using a photodiode array detector (HPLC-PDA). We also developed a rapid, simple, and low-cost thin layer chromatography (TLC) method for screening/detection of basic colorants and a liquid chromatography/mass spectrometry (LC/MS) approach for their identification.

The method is applicable to various processed foods such as fat-based food (curry paste and tandoori chicken), chili products (gochujang and chili sauce), and protein-based food (shrimp powder and powder soup).

## Experimental

### Reagents and chemicals

All solutions were prepared with ultrapure Milli-Q water (Milli-Q, Milford, MA), which was used for preparing the aqueous mobile phase. Ammonium acetate, sodium hydroxide, ammonium formate, sodium sulfate, hydrochloric acid, tetrahydrofuran (stabilizer free) (THF), ethanol (EtOH), and acetic acid were purchased from Wako Pure Chemical Industry, Ltd. (Osaka, Japan); 2-butanone, ethyl acetate, and hexane were purchased from Kanto Chemical Co., Inc. (Tokyo, Japan). The high-performance liquid chromatography (HPLC) grade organic solvents methanol and acetonitrile were supplied by Merck (Darmstadt, Germany).

RB (purity 98.3%) was purchased from Wako Pure Chemical Industry, Ltd., whereas AO (purity 90.8%) and PA (purity 94.4%) were purchased from Chroma Technology Corp. and Acros Organics (Geel, Belgium), respectively. A saturated NaCl solution containing 0.1 mol/L NaOH was prepared by dissolving 4 g of NaOH in 1 L of the solution. A 1.6 mol/L ammonium formate solution (pH 2.5) was prepared by dissolving 10 g of ammonium formate in 50 mL of water and subsequently adjusting the pH to 2.5 with formic acid. Water was then added to the solution to obtain a 100 mL final solution volume.

### Preparation of standard solutions

Stock standard solutions of RB, AO, and PA were prepared by dissolving each standard compound in methanol in a volumetric flask at a concentration of 1 mg/mL. The stock standard solutions were further diluted with methanol to give standard solutions for the recovery tests (with concentrations of 250 and 25 *μ*g/mL). For the calibration curves, the stock solutions were diluted with a 1% acetic acid solution containing methanol to give five working standard solutions for analysis (with concentrations of 0.05, 0.1, 0.5, 1, and 2 *μ*g/mL).

### Preparation of sample solutions from food

All food samples, that is, curry paste, chili sauce, gochujang (hot pepper paste), tandoori chicken (roasted chicken prepared with yogurt and spices), shrimp powder, and powder soup were obtained from a market in Tokyo. Solid samples were finely cut or homogenized. A 5 g portion was accurately weighted and dissolved in 20 mL of a solution containing 0.1 mol/L HCl:EtOH (1:2). The sample solution was then shaken for 1 min, and ethyl acetate (20 mL) was subsequently added with further agitation for 1 min. The solution was finally centrifuged at 3000 rpm (1500–2000*g*) for 1 min, and the supernatant was collected into a separatory funnel. The same treatment was performed two times on the residual precipitates, and the supernatant liquid was collected into the separatory funnel for ethyl acetate extraction. After adding 1 mL of an NaOH solution (2.5 mol/L) to the ethyl acetate extraction layer (except for the shrimp powder), 50 mL of a saturated NaCl solution (containing 0.1 mol/L NaOH) was added to the ethyl acetate extraction layer in the separatory funnel. The mixture was then shaken, and the latter layer was removed. After adding 40 mL of hexane and 20 mL of 0.1 mol/L HCl to the residual ethyl acetate extraction layer in the separatory funnel and shaking the mixture again, the basic colorants were extracted to the latter layer and collected in a 100 mL measuring flask. A further amount of HCl (0.1 mol/L, 20 mL) was added to the remaining layer upon agitation, and this layer was also collected (together with the rest) into the 100 mL measuring flask. Water was then added to the flask to obtain a final solution volume of 100 mL.

A 20 mL aliquot of the prepared solution was carefully taken, and its pH value was adjusted to 10–12 using a 2.5 mol/L NaOH solution. This mixture was employed to precondition the Oasis HLB column using 10 mL of methanol and water. The column was washed with 10 mL of Mill-Q water and eluted using 4 mL of 1% acetic acid in methanol. A further amount of acetic acid containing methanol was added to the mixture to obtain a final volume of 5 mL as a sample solution for HPLC experiments.

### Recovery tests and method validation

Recovery tests were performed to evaluate the accuracy of the developed method. A small amount of the standard solutions (0.1 mL, 25 *μ*g/mL) was added to 5 g of shrimp powder, powder soup, curry paste, chili sauce, gochujang, and tandoori chicken (finely cut) in the absence of PA, AO, and RB. The samples were kept at room temperature for 30 min and then treated as described in the section Preparation of sample solutions from food.

Calibration curves were prepared with the PA, AO, and RB standard solutions at concentrations of 0.05–50 or 100 *μ*g/mL to examine the linearity of the calibration curves. Intraday precision (RSD_r_) and interday precision (RSD_R_) were assessed by analyzing duplicates of a curry paste spiked with PA, AO, and RB (0.5 *μ*g/g) during a day and on five different days, respectively. The limits of detection (LOD) and quantification (LOQ) for PA, AO, and RB were estimated by the signal-to-noise S/N > 3 and S/N > 10 ratios of each peak in the standard solutions, respectively.

### HPLC analysis

The LC system consisted of a Hewlett Packard 1100 series (Agilent Technologies, Palo Alto, CA) containing a G1315A PDA detector (monitored at 550 nm for PA and RB, and 450 nm for AO), the L-column (octadecylsilane (ODS); i.d.: 4.6 mm × 150 mm; particle size: 5 *μ*m; pore size: 12 nm; Chemicals Evaluation and Research Institute, Tokyo, Japan), and a column heater set at 40°C. The mobile phase consisted of 20 mmol/L ammonium acetate, brought to pH 4.5 by dropwise addition of acetic acid (mobile phase A) and acetonitrile (mobile phase B). The gradient conditions were as follows: (1) a linear gradient from 20% to 60% mobile phase B (for 15 min) and (2) isocratic elution at 60% mobile phase B (for 5 min). The injection volume was 20 *μ*L and the flow rate was 1.0 mL/min. The apparatus was controlled (and the data were collected and analyzed) by using the Agilent Chemstation software.

### TLC analysis

TLC experiments were performed on 20 cm × 20 cm TLC RP-18 plates from Merck, which were cut into segments of 10 cm × 10 cm. Exactly 2 mL of the sample solutions was taken for HPLC, purged with nitrogen gas at room temperature, and concentrated to 0.2 mL. The sample solutions for TLC were spotted with 5–20 *μ*L (∼5–20 ng of each basic colorant), and standard solutions (5 *μ*g/mL) of the basic colorants were spotted with 1–4 *μ*L (∼5–20 ng of each basic colorant) using a 5 *μ*L capillary glass tube at 20 mm from the bottom of the plate.

The plates were developed up to 7 cm in a saturated developing chamber (Camag, Muttenz, Switzerland) for 10 cm × 10 cm plates. The developing solvents were 2-butanone–methanol–5 w/w% Na_2_SO_4_ (1:1:1, v/v/v) (solvent system A) and 2-butanone–methanol–1.6 mol/L ammonium formate (pH 2.5) (7:2:7, v/v/v) (solvent system B). After development, the plates were dried at room temperature and observed under white light for PA, AO, and RB, as well as at 254 nm for RB and 366 nm for AO and RB. The plates were documented by using a TLC visualizer (Camag).

### LC/MS analysis

LC analysis was performed by using a HPLC–electrospray ionization-MS (HPLC–ESI-MS) instrument from Shimadzu (LCMS-2010; Shimadzu, Kyoto, Japan). Chromatographic separation was performed on a reversed-phase HPLC L-column ODS (i.d.: 2.1 mm × 150 mm; particle size: 5 *μ*m; pore size: 12 nm). The mobile phase consisted of 20 mmol/L ammonium acetate (pH 4.5) (mobile phase A) and acetonitrile (mobile phase B). The gradient conditions were as follows: (1) a linear gradient from 20% to 60% mobile phase B (for 15 min) and (2) isocratic elution at 60% mobile phase B (for 5 min). The flow rate was 0.2 mL/min and the injection volume was 5 *μ*L; the column oven was maintained at 40°C. The sample solution for HPLC was injected and analyzed in the ESI (+) mode with selected ion monitoring (SIM) using selected ion masses of *m*/*z* 288[PA–Cl], *m*/*z* 268[AO–Cl], and *m*/*z* 443[RB–Cl–H] for LC/MS detection.

## Results and Discussion

### Optimization of the sample solution preparation

**Figure 2:**
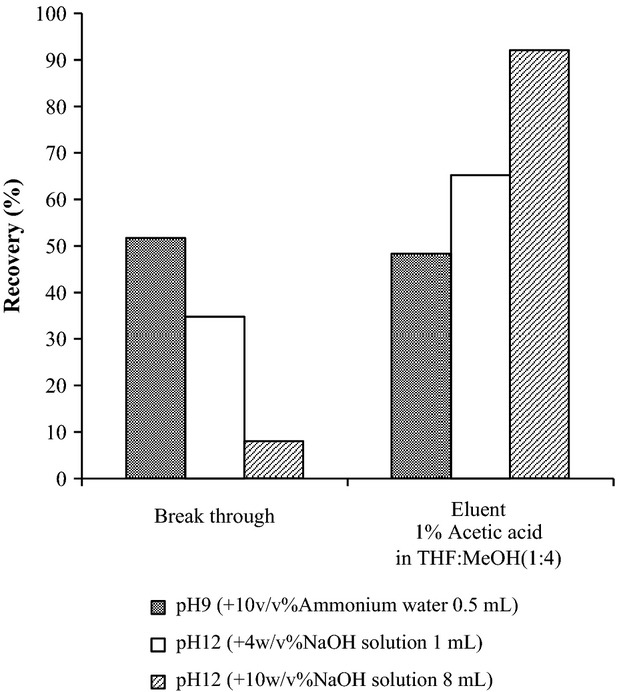
Recovery (%) of PA from an Oasis HLB under different pH conditions.

To optimize extraction conditions, we examined the extraction time and the number of extractions using 0.1 mol/L HCl:ethanol (1:2) and ethyl acetate as the extraction solvent. As shown in Figure 2, the recovery rate of RB in chili powder was above 90% after three extractions.

To remove impurities from the extracted solution, we applied the salt-out method using a saturated NaCl solution containing 0.1 mol/L NaOH (basified saturated NaCl solution). This procedure helped in removing impurities in the solution extracted from chili powder.

We considered that impurities were effectively removed from the extracted chili-powder sample using a saturated NaCl solution containing 0.1 mol/L NaOH. When such a solution is added to the ethyl acetate extraction layer, the extracted solution is normally expected to have a pH of 9–11. However, in the case of the chili sauce, the pH appeared to be below 9. This observation indicates a loss in the amount of PA in the ethyl acetate layer because of the influence of the matrix. The presence of a thickener or vinegar in the chili sauce may cause an amount of PA to remain in the NaCl/NaOH layer. To prevent this loss, we added NaOH (1 mL, 2.5 mol/L) to the extracted solution in the ethyl acetate layer before adding saturated NaCl containing NaOH. Apparently, PA could be effectively extracted in the ethyl acetate layer using the proposed procedure. From this result, we can conclude that when the pH of the ethyl acetate extraction layer is not sufficiently alkaline from the effect of the food matrix alone, it is necessary to add a 2.5 mol/L NaOH solution adjust the pH to alkaline.

### Optimization of the clean-up process on solid-phase extraction columns

Many solid-phase extraction columns such as octadecyl silica (ODS; Gagliardi et al. 1996), styrene–divinylbenzene polymeric surfaces (Strata-X, Strata-SCX, Oasis HLB, Sepabeads® SP70; Mitrowska et al. 2005; Lee et al. 2006; Chiang et al. 2011; Soylak et al. 2011), alumina and strong cation exchange columns (Halme et al. 2004), immune affinity columns (Xie et al. 2013), and polyamide (Dixit et al. 2011) have been used to clean-up basic synthetic dyes from foods, cosmetic products, and tissues.

Chiang et al. (2011) and Soylak et al. (2011) reported that stylene–divinylbenzene polymers are effective in cleaning basic dyes such as RB and malachite green from foods. Therefore, we attempted to clean-up the basic dyes from the prepared solutions using a cartridge containing a stylene–divinylbenzene polymer (Oasis HLB). We found that the basic dyes, except PA, could be retained in the cartridge, and we believe that the breakthrough of PA could be because of its positive charge. Therefore, we added an alkaline solution to the prepared solution after dilution with distilled water to neutralize the basic dye charge. As expected (see Fig. 2), we found that PA could then be retained in the cartridge and was recovered substantially from the sample.

The prepared solution was diluted three times with water to reduce the effects of the organic solvent. RB and AO were retained in the Oasis HLB, whereas PA was not. We therefore examined the effect of pH of the eluent on the retention of PA.

To achieve this, we adjusted the pH of a diluted solution prepared solution from chili sauce spiked with PA to 9 using 10% ammonium water, and to pH 12 using 0.5 mol/L NaOH and 8 mL of 2.5 mol/L NaOH solutions. The sample was then applied to the Oasis HLB, and a 1 mL fraction was collected. As shown in Figure 2, in the case of the diluted solutions prepared by using 10% ammonium water (pH 9) and 0.5 mol/L NaOH (pH 12), the pH was slightly acidic so that 30%–50% of the PA was not retained by the cartridge. On the other hand, the PA contained in the diluted solution using 2.5 mol/L NaOH (pH 12, 8 mL) solution was retained by the cartridge with less breakthrough. An improved PA recovery of 90% was achieved by elution with 1% acetic acid in methanol:THF (4:1).

**Figure 3:**
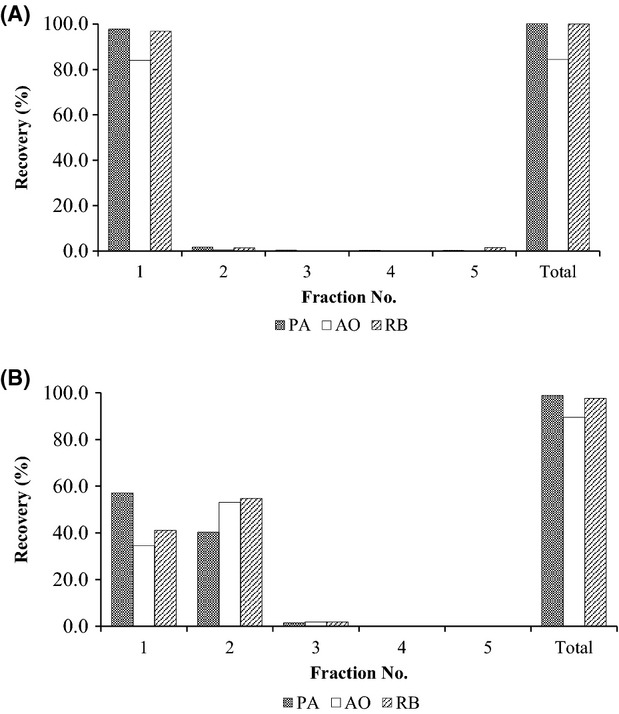
Recovery (%) of PA, AO, and RB from an Oasis HLB using different eluents: 1% acetic acid in THF:MeOH (1:4) (A) and 1% acetic acid in MeOH (B).

This solution was tested as an eluent, and more than 90% of RB, AO, and PA was effectively eluted in the first 1 mL (Fig. 3A). However, as shown in Figure S1 of the chromatogram in the HPLC analysis of basic colorants after elution from the cartridge using 1% acetic acid in methanol:THF (4:1), the peaks corresponding to the basic colorants are broad, and the sensitivity to AO is low, even if the pH is changed from 3.5 to 6.5 to optimize the HPLC conditions. Since THF is generally unstable—it affects the peak shape in the HPLC analysis—we replaced the 1% acetic acid in methanol:THF (4:1) with 1% acetic acid in pure methanol eluent. As shown in Figure 3B, the shapes of the colorant peaks in the HPLC chromatogram improved, and the sensitivities were higher than those obtained by using THF as an eluent, although 1–2 mL of eluent was necessary to effectively elute all the color from the cartridge. Therefore, 1% acetic acid in methanol was used as an appropriate eluent for the purification of colorants from the cartridge.

### Optimization of HPLC conditions

According to previous reports on synthetic food colorant analyses by HPLC or LC/MS, an aqueous ammonium acetate solution (solvent A) and acetonitrile (solvent B) were used as the mobile phase for the gradient conditions in HPLC analysis (Suzuki et al. 2007). In this study, we tried to optimize the pH of solvent A.

**Figure 4:**
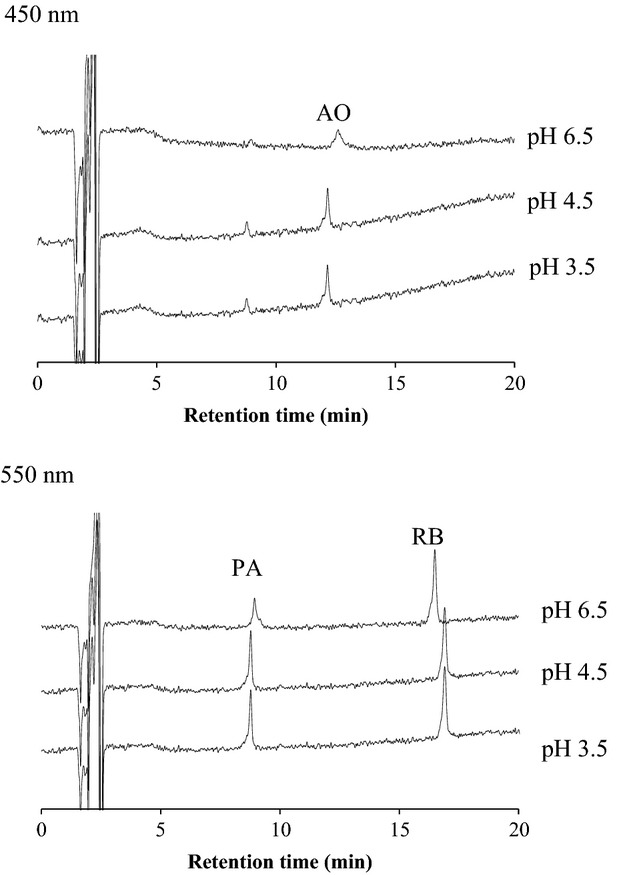
HPLC chromatograms (at 450 and 550 nm) of standard solutions of PA, AO, and RB (0.1 *μ*g/mL) at different pH values (pH 6.5, 4.5, and 3.5).

As shown in Figure 4, the AO and PA peaks are broad, and the S/N ratios obtained by using solvent A at pH 6.5 (i.e., 5 for AO and 9.5 for PA) are lower than those obtained at pH 4.5 (8 for AO and 17 for PA) or pH 3.5 (5 for AO and 8 for PA), suggesting that the sensitivities for the determination of AO and PA using solvent A at pH 6.5 are lower than those at pH 4.5 or 3.5. In addition, in the case of RB, the retention time (RT) using solvent A at pH 3.5 is longer than that at pH 4.5 (Fig. 4), suggesting that the analysis of RB will take longer in the former case than in the latter one. The sensitivities of AO and PA at pH 4.5 are similar to those at pH 3.5, and therefore, we used 10 mmol/L ammonium acetate (at pH 4.5) as solvent A for the HPLC analysis of basic colorants.

### Validation of the method

The analytical method developed herein was validated by determining its linearity, LOD, LOQ, trueness (by recovery tests), and precision. The calibration curve for PA exhibits linearity at the concentration of 0.05–50 *μ*g/mL, whereas those for AO and RB are linear at 0.05–100 *μ*g/mL. The regression coefficients were greater than 0.999 in all cases (PA, AO, and RB).

The LOD and LOQ were determined by using standard solutions. The LODs based on three times the S/N ratio were 0.0125 *μ*g/g for PA, 0.05 *μ*g/g for AO, and 0.0125 *μ*g/g for RB. The LOQs based on ten times the S/N ratio were 0.05 *μ*g/g for PA, 0.125 *μ*g/g for AO, and 0.025 *μ*g/g for RB. The confirmatory LOD for the three basic colorants based on visual evaluation of the PDA spectra was estimated to be 0.025 *μ*g/g.

The accuracy and precision of the method were evaluated by recovery tests. Standard solutions of PA, AO, and RB were spiked with shrimp powder, powder soup, curry paste, chili sauce, gochujang, and tandoori chicken at a final colorant concentration of 0.5 *μ*g/g. Table 1 shows the recoveries and relative standard deviations (RSDs) obtained by the developed analytical method. The recoveries and RSDs for PA, AO, and RB ranged from 70.2% to 102.8%, and from 0.8% to 8.0%, respectively, except for the shrimp powder, where the values ranged from 51.7% to 75.2% and 2.3% to 26.4%, respectively. The lower recoveries and precision values observed for the shrimp powder could be because of losses in PA, AO, and RB during purification using a cartridge, which involves precipitation induced by adding 1 mL of a 2.5 mol/L NaOH solution to the ethyl acetate extraction layer. Thus, to prevent precipitation, we did not add the NaOH to the solution prepared from shrimp powder; the prepared solution was purified using Oasis HLB. Consequently, the recoveries were improved to 87.8% for PA, 76.8% for AO, and 95.2% for RB.

**Table 1:** Recoveries of pararosaniline (PA), auramine O (AO), and rhodamine B (RB) from spiked food matrices (shrimp powder, powder soup, curry paste, chili sauce, gochujang, and tandoori chicken)

	Recovery (%)^1^		
	PA	AO	RB
Tandoori chicken	102.4 ± 0.8	92.7 ± 5.7	91.3 ± 1.1
Gochujang	102.8 ± 3.3	92.6 ± 9.7	93.6 ± 0.9
Chili sauce	85.4 ± 8.0	92.7 ± 5.8	95.9 ± 1.9
Curry paste	86.2 ± 2.6	85.4 ± 6.3	92.8 ± 1.7
Powder soup	88.1 ± 3.2	70.2 ± 1.7	99.0 ± 3.6
Shrimp powder^2^	87.8 ± 3.9	76.8 ± 3.0	95.2 ± 2.9

1 Mean ± SD.

2 Recoveries from the shrimp-powder sample solution, which was prepared without adding NaOH.

The reproducibility of the results was assessed by determining both the RSD_r_ and the RSD_R_ of the recovery tests by spiking standard solutions of PA, AO, and RB with concentrations of 0.5 *μ*g/g of each basic colorant in the curry paste. The RSD_r_ values ranged from 1.7% to 4.5%, and the RSD_R_ values ranged from 3.7% to 7.7% (Table 2).

**Table 2:** Intraday (RSD_r_) and interday (RSD_R_) precision data for curry paste spiked with PA, AO, and RB

				Precision	
Analyte	Spiked level (μg/g)	Found (μg/g)^1^	Recovery (%)	Intraday RSD_r_	Interday RSD_R_
PA	0.50	0.40 ± 0.03	80.0	2.9	7.7
AO	0.50	0.43 ± 0.02	85.4	4.5	3.7
RB	0.50	0.49 ± 0.02	97.9	1.7	4.1

PA, pararosaniline; AO, auramine O; RB, rhodamine B.

1 Mean ± SD.

**Figure 5:**
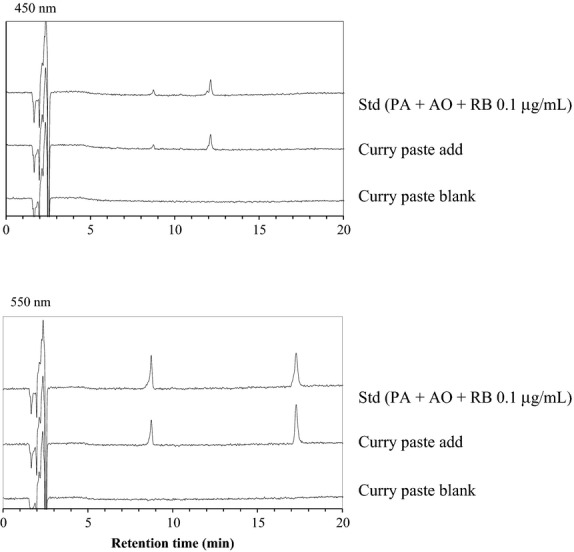
HPLC chromatograms (at 450 and 550 nm) of the standard solutions of PA, AO, and RB (0.1 *μ*g/mL), a blank solution, and a sample solution from curry paste.

Typical chromatograms of the analysis of PA, AO, and RB in recovery tests using the curry paste are shown in Figure 5. The peaks obtained for the basic colorants were well separated, with RT of 8.5 min for PA, 12 min for AO, and 17.5 min for RB. The PDA spectrum of the sample solution for the HPLC experiments agrees well with that of the standard solution. As shown in Figure S2A–E, we obtained well-separated HPLC chromatograms of the analysis of PA, AO, and RB in the recovery tests using tandoori chicken, gochujang, chili sauce, powder soup, and shrimp powder.

### TLC

**Figure 6:**
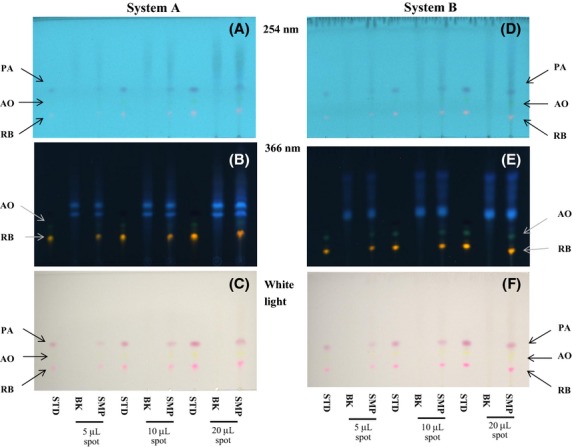
TLC chromatograms of a standard solution (STD), a blank solution (BK), and a sample solution (SMP) from curry paste at 254 and 366 nm, as well as under white light using the developing solvent systems A [2-butanone–methanol–5%Na_2_SO_4_ solution (1:1:1, v/v/v)] and B [2-butanone–methanol– 1.6 mol/L ammonium formate solution (pH 2.5) (7:2:7, v/v/v)].

To apply the prepared sample solutions in conventional TLC experiments and assess the detection limit for basic colorants by TLC, we performed TLC analysis using the prepared sample solutions (5–20 *μ*L, ∼5–20 ng of basic colorant) in the recovery tests described in the section Validation of the method. Typical TLC chromatograms obtained for PA, AO, and RB in recovery tests using a curry paste are shown in Figure 6A–F. As shown in Figure 6C and F, PA, AO, and RB were separately detected as red, yellow, and pink spots in systems A and B by spotting more than 5 *μ*L of sample solution under a white light. However, it was difficult to visually detect the AO spot under the white light because of its yellow color. AO and RB were visually detected as fluorescent yellow and orange spots at 366 nm, separated from the fluorescent blue spots from impurities of the curry paste in systems A and B (Fig. 6B and E). A spot of AO was clearly detected at 366 nm in system B. Spots of PA, AO, and RB were also visually detected at 254 nm when more than 5 *μ*L of sample solution was used in systems A and B. However, the fluorescence intensities of the AO and RB spots, as well as the visual intensity of the PA spot, were much lower than those observed under white light and at 366 nm. As shown in Figure S3A–E, we observed similar TLC of the sample solution from tandoori chicken, gochujang, chili sauce, powder soup, and shrimp powder.

### LC/MS

**Figure 7:**
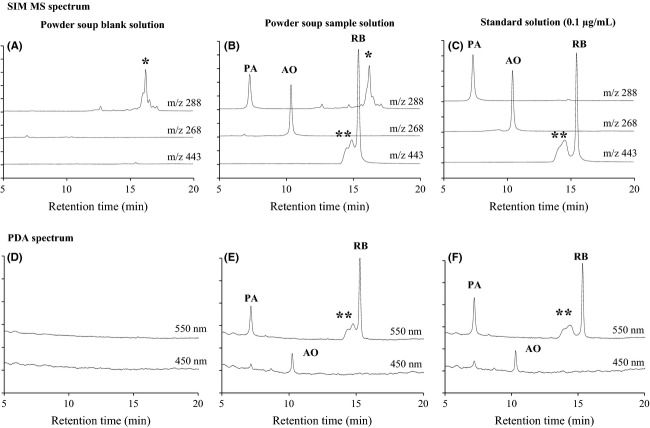
LC/MS SIM chromatograms of sample solutions from a powder soup spiked with PA, AO, and RB (each spiked level is 0.5 *μ*g/g) and PDA chromatograms at 450 and 550 nm. The symbol “*” represents an artifact from the processed food and “**” represents an artifact from RB.

To correctly identify PA, AO, and RB in sample solutions prepared from processed foods for the regulation of unauthorized basic colorants, a confirmation by LC/MS analysis is necessary. Therefore, we developed a qualitative LC/MS method to identify the basic colorants, although the quantitative performance of the method was not assessed. Figure 7A–F shows typical LC/MS SIM and HPLC-PDA chromatograms at 550 and 450 nm for the analysis of PA, AO, and RB in recovery tests using a powder soup. Peaks of the RT were detected at 8.5 min for PA, 11.7 min for AO, and 16.1 min for RB in the SIM (Fig. 7B and C) and HPLC-PDA (Fig. 7E and F) chromatograms of the standard solutions and sample solutions prepared from a powder soup spiked with basic colorants. As shown in Figure 7B, C, E, and F, interference peaks were detected in the case of RB before the RT peak, both in the SIM and HPLC-PDA chromatograms of the sample solution and the standard solution. These interference peaks appear to be derived from RB. Similarly, as shown in Figure S4, peaks for PA, AO, and RB were detected in the SIM and HPLC-PDA chromatograms of sample solutions prepared from other processed foods (i.e., chili sauce, curry paste, gochujang, tandoori chicken, and shrimp powder).

In the SIM chromatogram of a blank solution (Fig. 7A), multiple peaks were detected at approximately 16 min (*m*/*z* 288 for PA). However, the RT of these peaks was different from that of the PA standard solution, and since the peaks also appeared in the blank solution chromatograms of sample solutions prepared from other foods, we considered that they could probably be the impurities derived from the foods.

## Conclusions

We developed an HPLC method for the determination of unauthorized basic colorants in processed foods. The recoveries achieved by this procedure ranged from 70.2% to 102.8%. The HPLC method offers a way to reduce interferences in fat-based food matrices (curry paste and tandoori chicken), water-soluble substances (chili color in gochujang and chili sauce), or protein-based products (shrimp powder and powder soup). This study shows that the proposed method is a simple and reliable way to determine unauthorized basic colorants such as PA, AO, and RB in processed foods. Furthermore, a TLC method for the screening/detection of the colorants, as well as an LC/MS approach to identify them, was also developed. The LC/MS approach was developed for qualitative purposes and has not been validated quantitatively. Further studies to determine basic colorants, both quantitatively and qualitatively, in processed foods by LC/MS would be necessary. We believe that these methods could be very useful for monitoring unauthorized basic colorants in inspection centers or quarantine laboratories in many countries.

## Supporting Information

**Figure S1.** HPLC chromatograms of standard solutions of PA, AO, and RB (0.1 *μ*g/mL) at different pH values (6.5, 4.5, and 3.5) in 1% acetic acid in THF:MeOH (1:4). **Figure S2.** HPLC chromatograms (at 450 and 550 nm) of the standard solutions of PA, AO, and RB (0.1 *μ*g/mL), a blank solution, and a sample solution from curry paste. **Figure S3.** TLC chromatograms of a standard solution (STD), a blank solution (BK), and a sample solution (SMP) from tandoori chicken, gochujang, chili sauce, powder soup, and shrimp powder at 254 and 366 nm, as well as under white light using the developing solvent systems A [2-butanone–methanol–5% Na_2_SO_4_ solution (1:1:1, v/v/v)] and B [2-butanone–methanol 1.6 mol/L ammonium formate solution (pH 2.5) (7:2:7, v/v/v)]. **Figure S4.** LC/MS SIM chromatograms of sample solutions from tandoori chicken, gochujang, chili sauce, curry paste, and shrimp powder spiked with PA, AO, and RB (each spiked level is 0.5 *μ*g/g).
