# Supplementary material for: A simple and rapid chromatographic method to determine unauthorized basic colorants (rhodamine B, auramine O, and pararosaniline) in processed foods
Source: Food Sci Nutr. 2014 Jun 2;2(5):547–56. doi: 10.1002/fsn3.127 (PMC4237484; doi:10.1002/fsn3.127)
Supplement: Supplementary file 1 [file fsn30002-0547-sd1.pdf]

Fig. 3 S1
Supplemental Figure

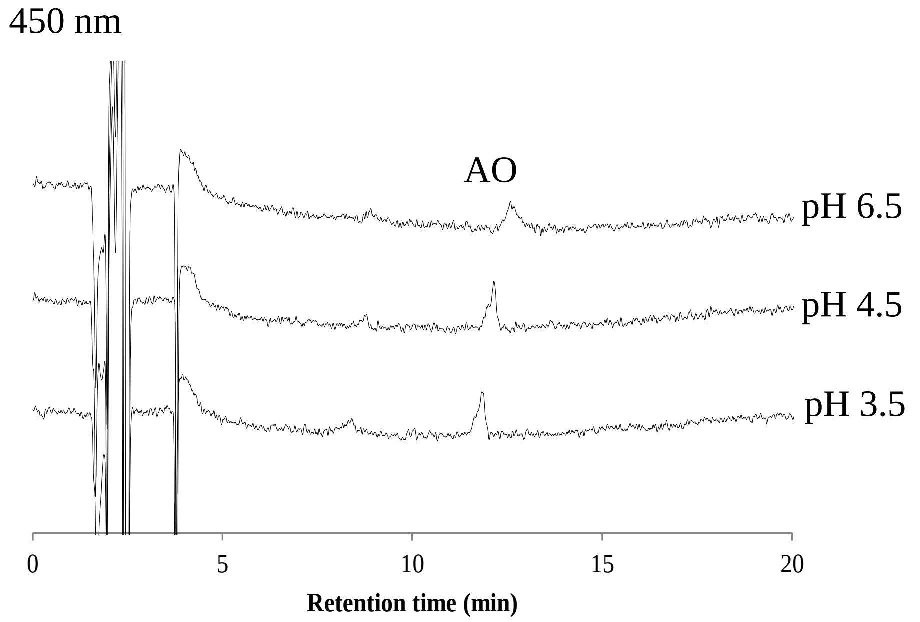

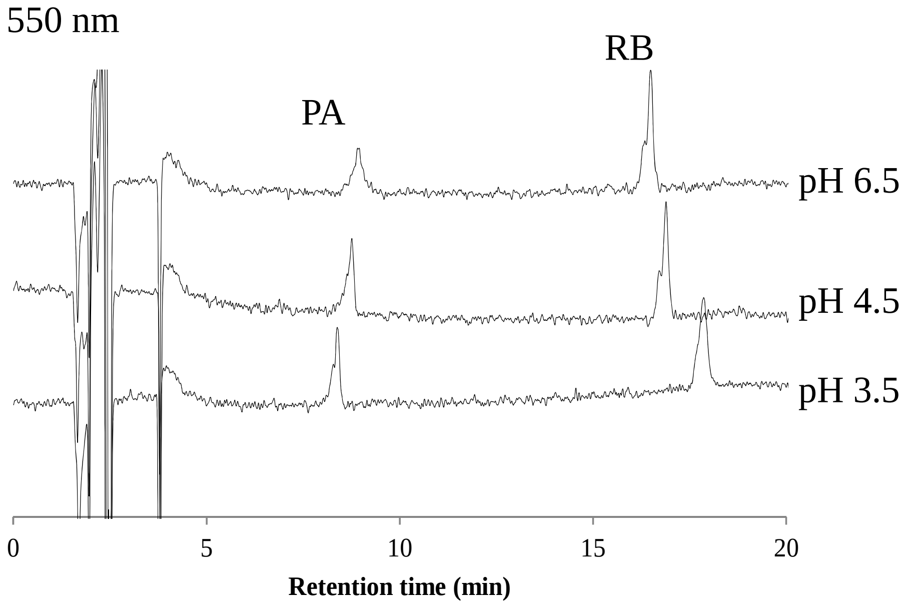

Fig. 5S
Supplemental Figure

(a) Tandoori chicken

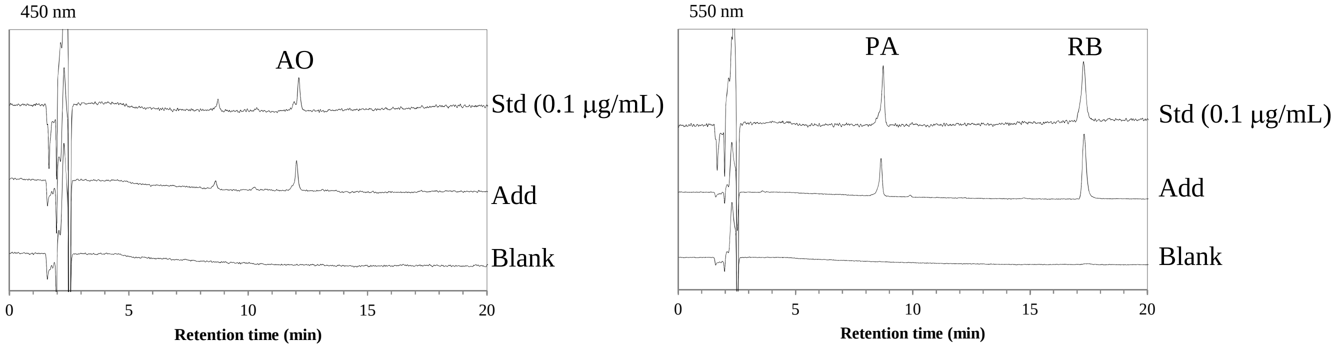

(b) Gochujang

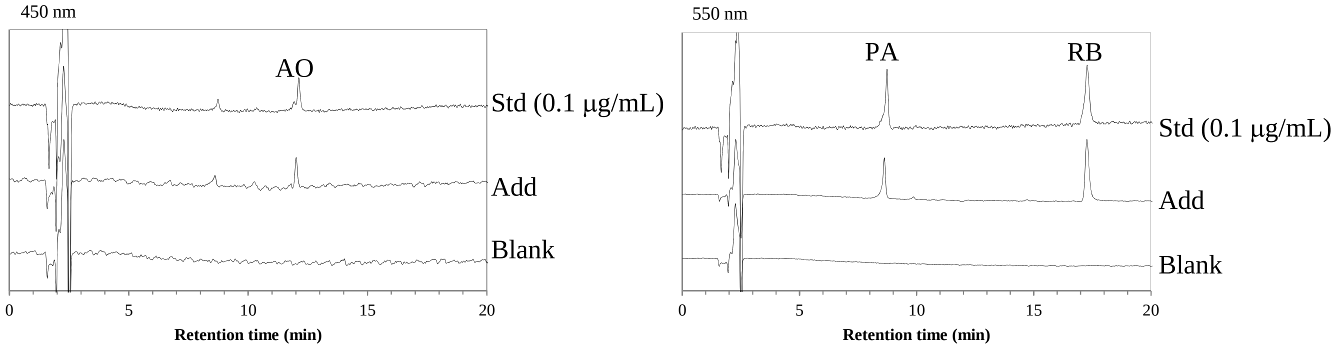

(c) Chili sauce

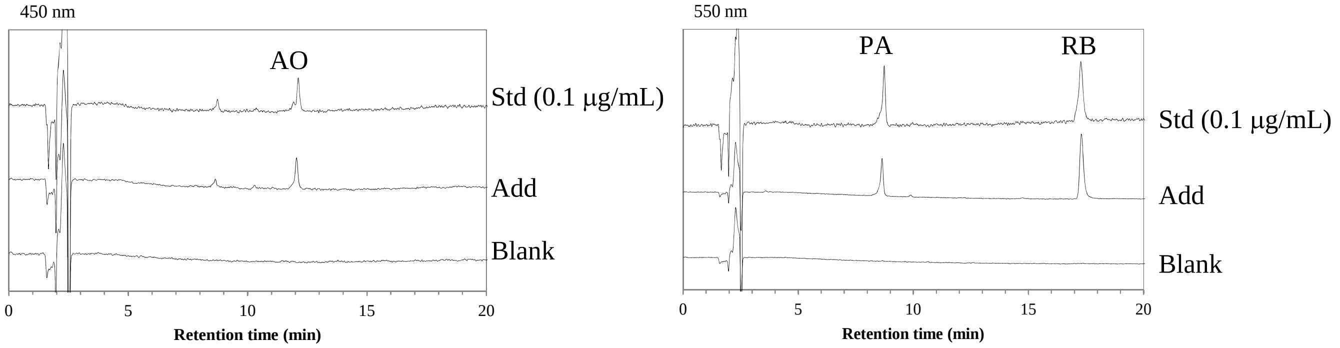

(d) Powder soup

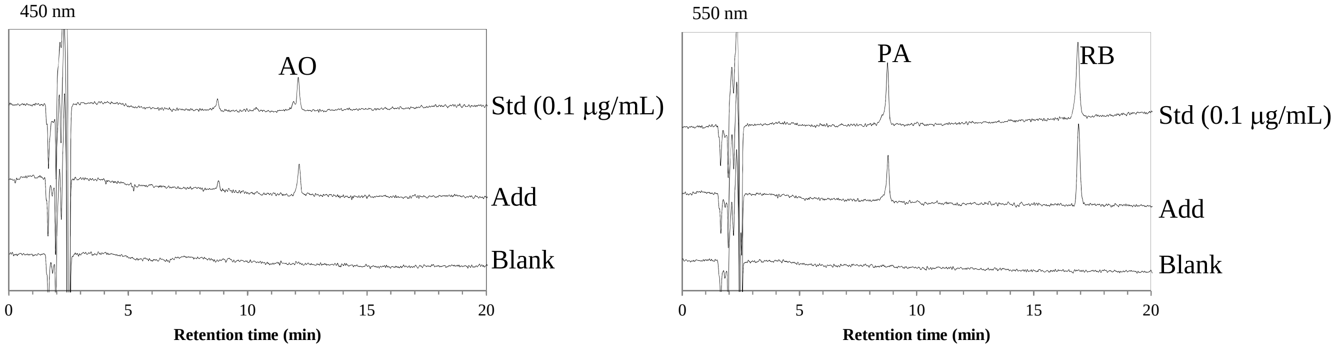

(e) Shrimp powder

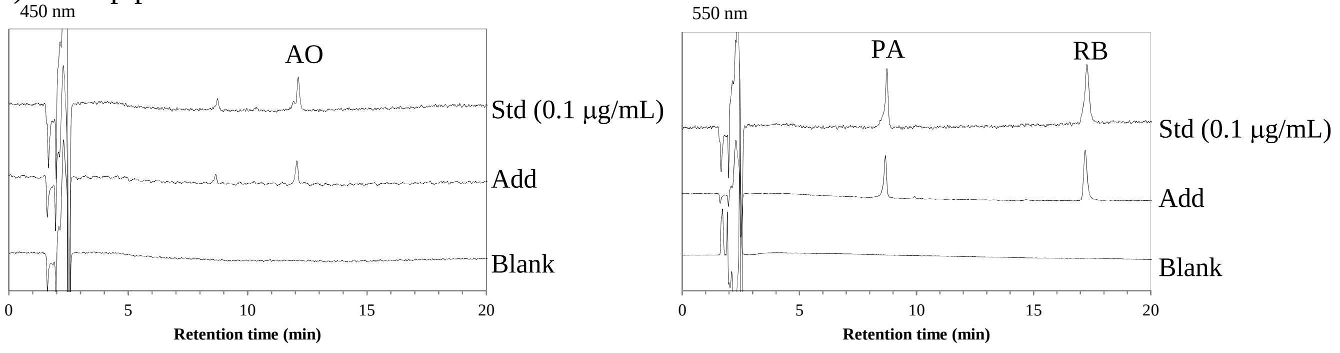

a) Tandoori chicken

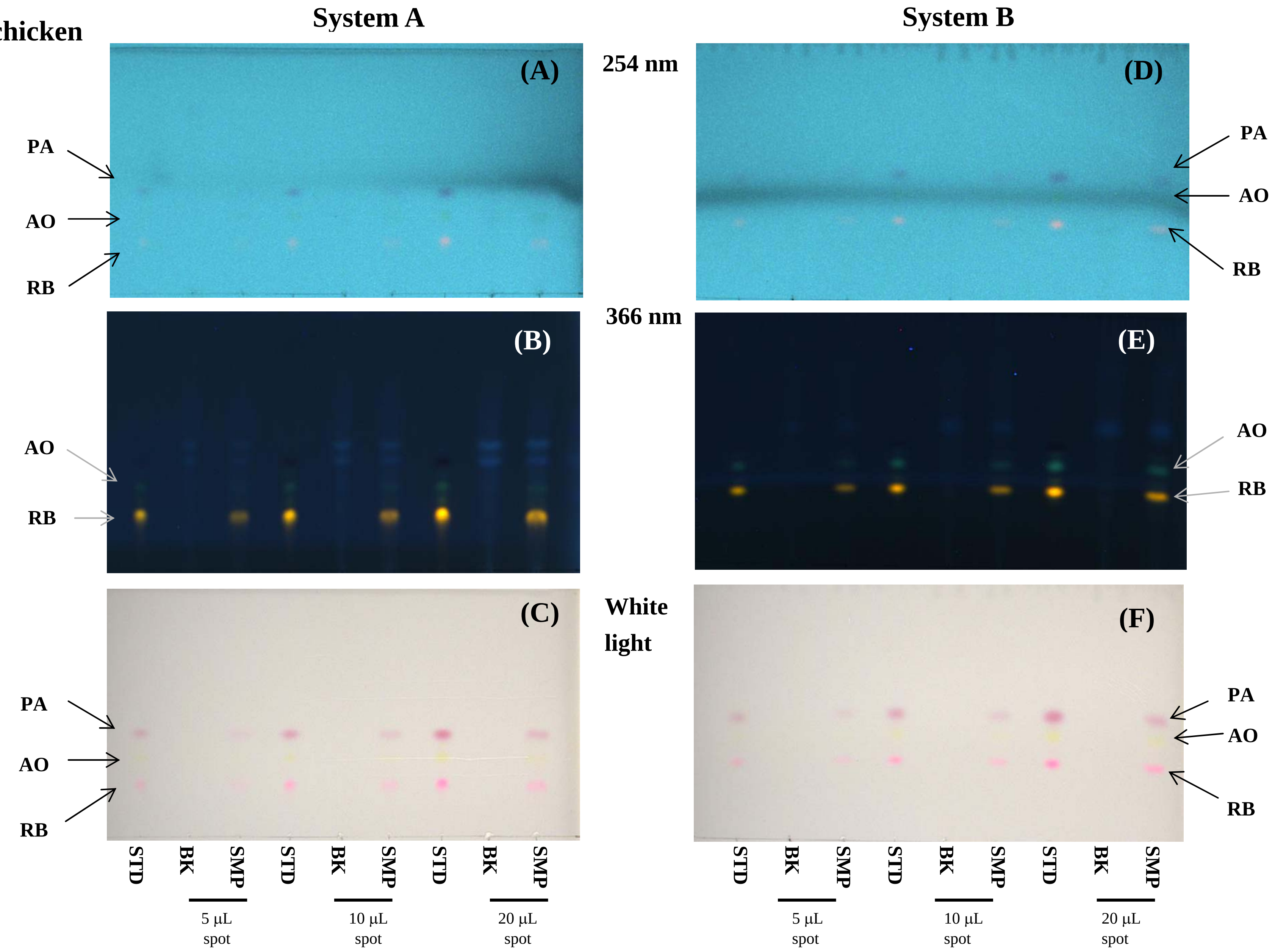

Fig. 6S
Supplemental Figure

b) Gochujang

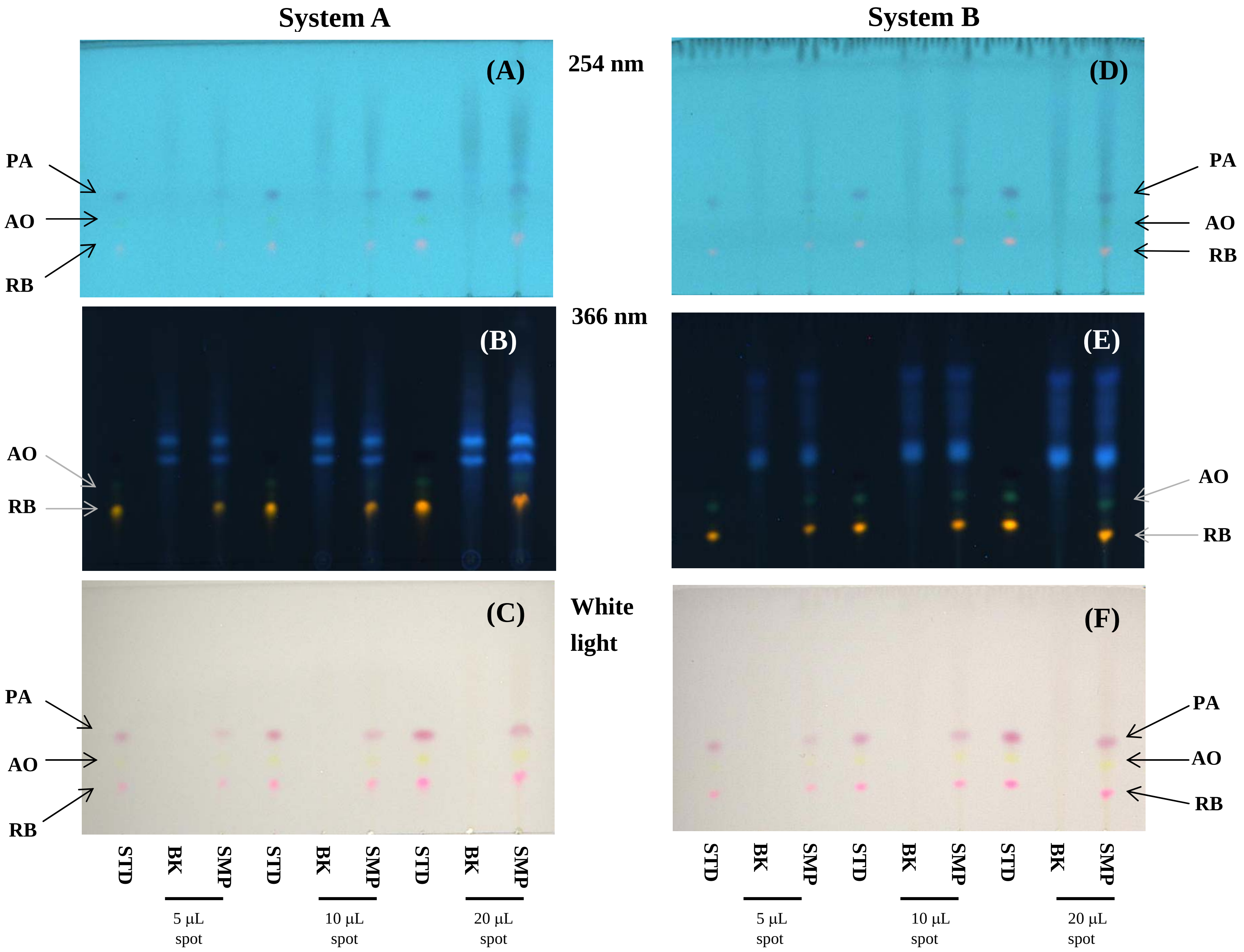

Fig. 6S
Supplemental Figure

c) Chili sauce

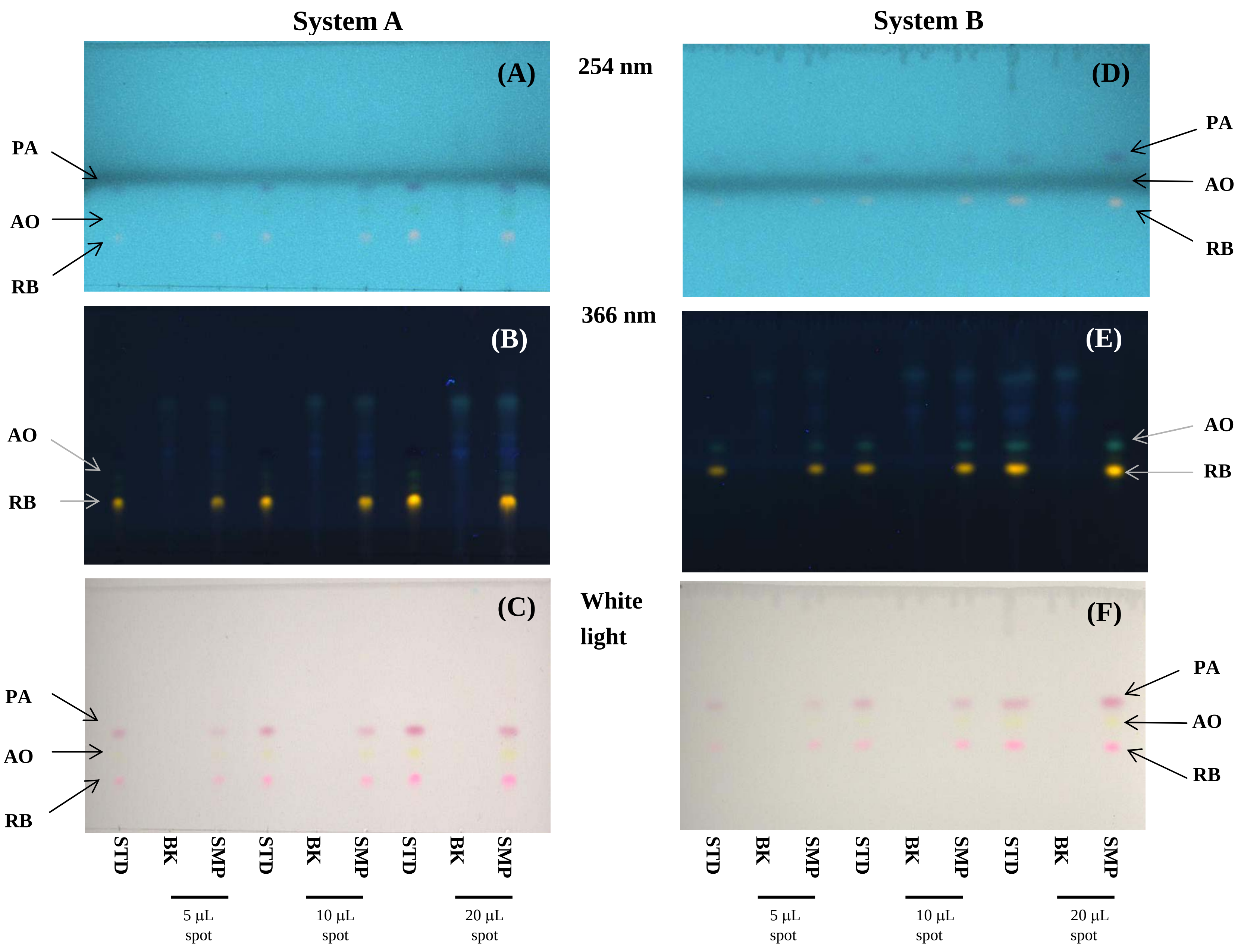

Fig. 6S
Supplemental Figure

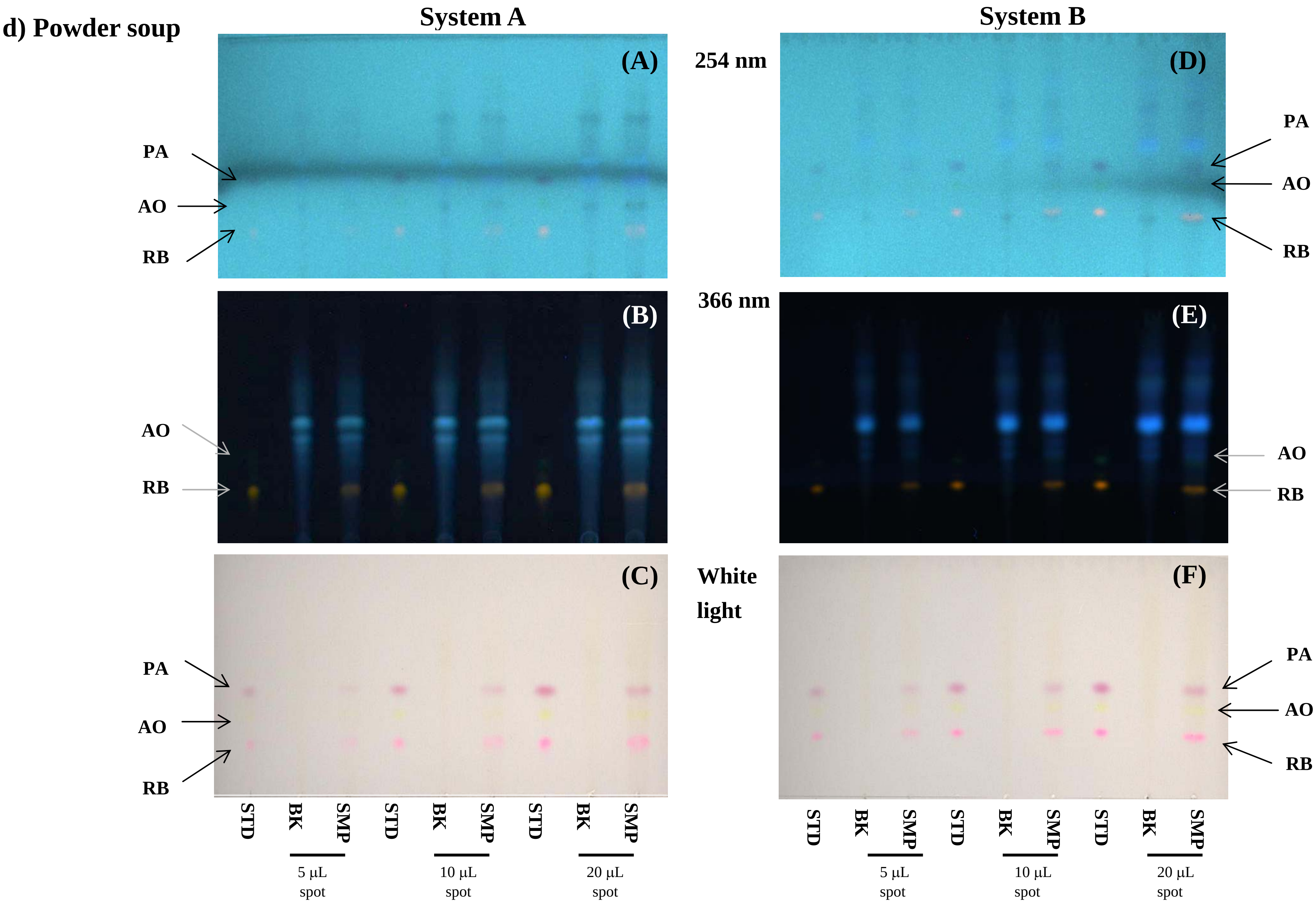

Fig. 6S
Supplemental Figure

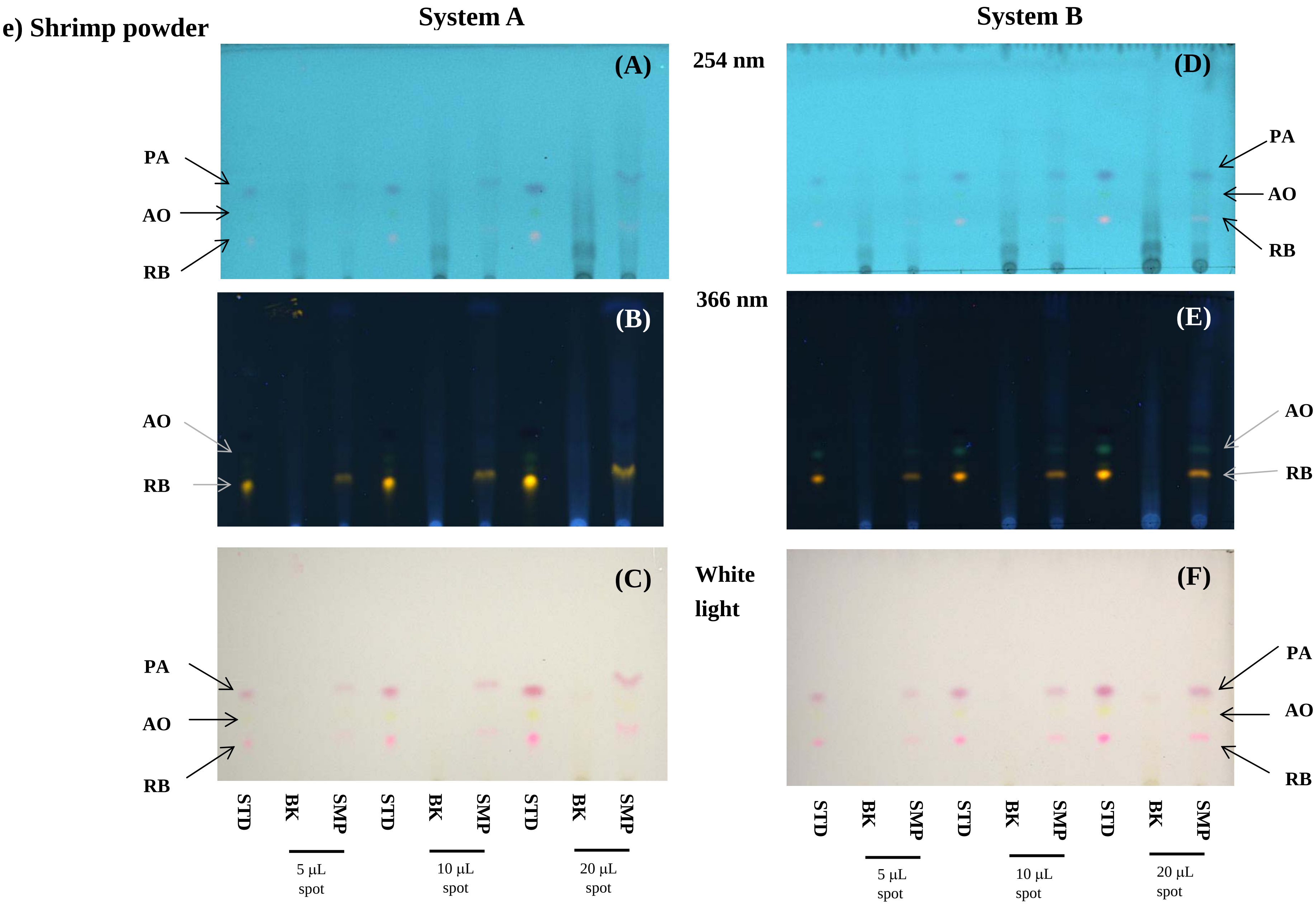

Fig. 6S
Supplemental Figure

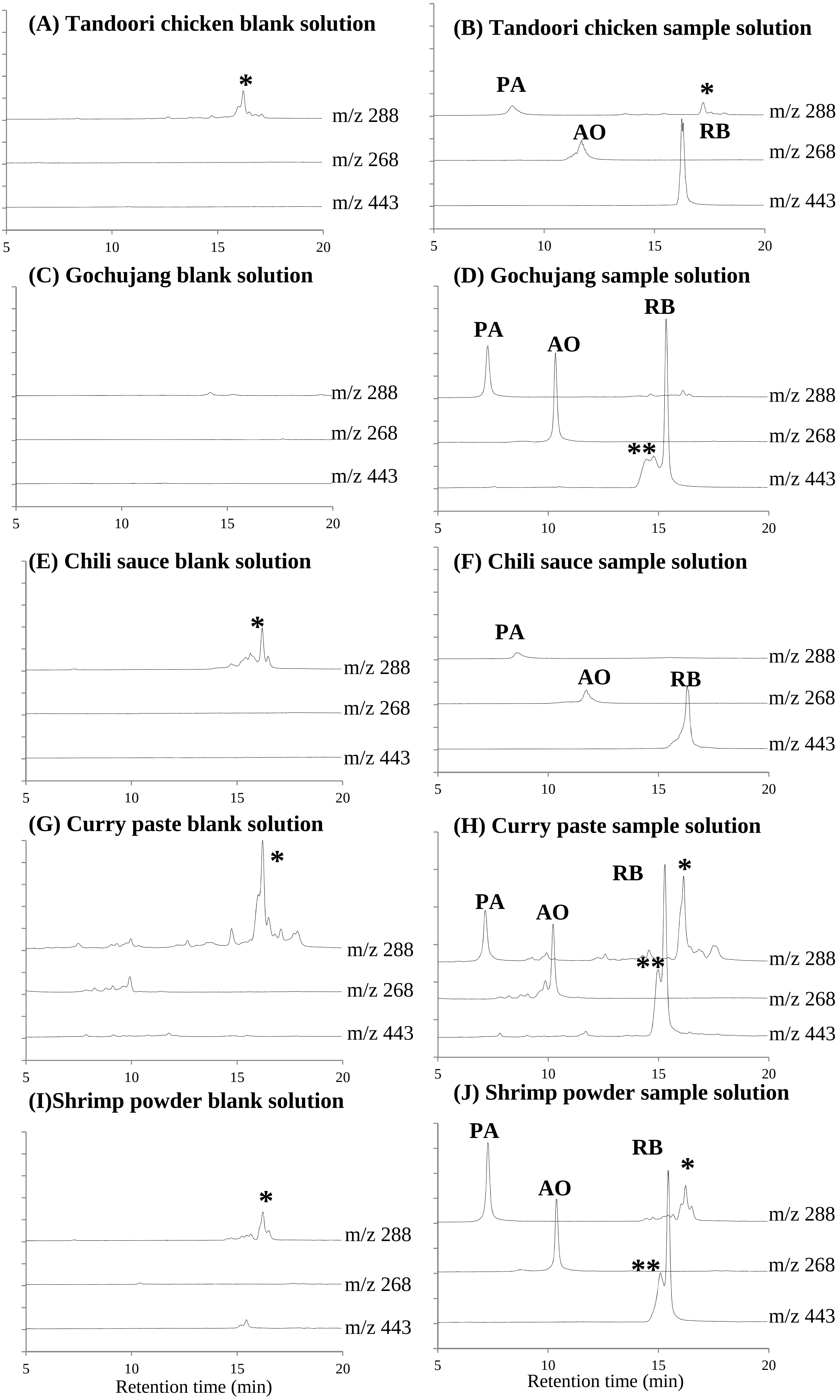

Fig. 7S
Supplemental Figure

Figure 3. S1

HPLC chromatograms of standard solutions of PA, AO, and RB (0.1 μg/mL) at different pH values (6.5, 4.5, and 3.5) in 1% acetic acid in THF:MeOH (1:4).

Figure 5S.

HPLC chromatograms (at 450 and 550 nm) of standard solutions of PA, AO, and RB (0.1 μg/mL), a blank solution, and a sample solution from curry paste.

Figure 6S.

TLC chromatograms of a standard solution (STD), a blank solution (BK), and a sample solution (SMP) from tandoori chicken, gochujang, chili sauce, powder soup and shrimp powder at 254 and 366 nm, as well as under white light using the developing solvent system A [2-butanone–methanol–5%Na_2SO_4 solution (1:1:1, v/v/v)] and B [2-butanone–methanol1.6 mol/L ammonium formate solution (pH 2.5) (7:2:7, v/v/v)].

Figure 7S.

LC/MS SIM chromatograms of sample solution from tandoori chicken, gochujang, chili sauce, curry paste and shrimp powder spiked with PA, AO, and RB (each spiked level is 0.5 μg/g)
